# Combined Lumbar-Sacral Plexus Block in High Surgical Risk Geriatric Patients undergoing Early Hip Fracture Surgery

**DOI:** 10.5704/MOJ.1511.004

**Published:** 2015-11

**Authors:** S Petchara, S Paphon, A Vanlapa, P Boontikar, K Disya

**Affiliations:** Department of Anesthesiology, Ramathibodi Hospital, Bangkok, Thailand; * Department of Orthopedics, Ramathibodi Hospital, Bangkok, Thailand

**Keywords:** Sole anaesthetic, combined lumbar and sacral plexus block, CLSB protocol, hip fracture surgery, peripheral nerve blockade

## Abstract

Objective: To evaluate the postoperative outcome after using combined lumbar and sacral plexus block (CLSB), as a sole anesthetic method in hip fracture (HF) surgery in highrisk geriatric patients.

Materials and Methods: A single-center retrospective study was conducted, between 2010 and 2012, on 70 elderly HF patients with American Society of Anesthesiologists grading III-IV who underwent early surgical intervention with our CLSB protocol. Perioperative data, outcome, and complications were recorded.

Results: Forty-eight patients (69%) had ongoing anticoagulant medication. Postoperatively, all patients were hemodynamically stable and awake. None of them required general anesthesia conversion. Minor anesthetic-related complications were found in nine patients. One patient (1%) died from sepsis due to pneumonia. Patients’ satisfactions were all rated as very good or excellent.

Conclusion: CLSB is an interesting anesthetic option in HF surgery, especially in high surgical risk geriatric patients. This method offers an excellent clinical efficiency and high patients’ satisfaction without serious complications.

## Introduction

Hip fracture (HF) is one of the major worldwide problems^1^that constitutes a significant mortality rate, ranging from 14-36% in the first year after injury^2,3^, and associated with profound temporary and sometimes permanent impairment of independence and quality of life in the geriatric population^4,5^. Generally, the treatment guideline recommends early surgical intervention within 48-72 hours after admission, either with general anesthesia (GA) or regional anesthesia (RA), to allow early ambulation, reduce mortality, and prevent postoperative morbidity such as pneumonia and pressure ulcer^6,7^. Recently, systemic review studies have demonstrated the benefits of using RA in HF operations as a significant reduction in mortality and morbidity such as venous thromboembolic complication, respiratory infection, and transfusion requirement^8-10^. Traditionally, the most common mode of RA in HF surgery is neuraxial anesthesia (NA), as spinal or epidural anesthesia. However, NA also has several disadvantages, such as intraoperative hypotension, urine retention, and epidural hematoma, which potentially lead to catastrophic consequences like myocardial infarction, stroke, and central nervous system infection^11^. Thus, NA might not be appropriate in some specific conditions, such as the high surgical risk geriatric patients and the patients who have concomitant anticoagulant or antiplatelet medications, which possess a higher risk of postoperative morbidity and mortality^12^. Recently, peripheral anaesthesia (PA), which block one or more peripheral nerves that supply the surgical field in the operated limb, has been proven to be an effective method for lower limb surgery^13^. Recently also, application of combined lumber and sacral plexus block (CLSB), has been introduced as a newer PA method in HF surgery with a comparable efficacy to NA^14^. Moreover, CLSB also demonstrated many advantages over NA, as better hemodynamic control in elderly patients and avoidance of the risk of NA-related complications^14^. However, there were only a few case reports related to using combined lumbar and sacral plexus block (CLSB) in high-risk geriatric patients^15,16^. Therefore, this study aimed to report the outcome after using CLSB in the high surgical risk geriatric patients who had HF and underwent early surgical intervention on the aspect of the effectiveness of CLSB in HF surgery, its safety, and complication related to this procedure.

## Materials and Methods

This was a single-center, retrospective chart analysis study with prior approval obtained from our institutional ethical review board, based on the Declaration of Helsinki. Eligible participants were patients diagnosed with hip fracture and who had received early surgical intervention under CLSB within 72 hours after admission in our institution from January 2010 to December 2012. The inclusion criteria were (1) age more than 60 years and sustained low-energy trauma, (2) newly diagnosed as closed femoral neck fracture or intertrochanteric fracture, and (3) being evaluated as high surgical risk, defined by having American Society of Anesthesiologist (ASA) physical status III or IV. The exclusion criteria were (1) operative delay more than 72 hours, (2) other pathological fracture such as metastatic fracture, and (3) previous fracture on the injured hip.

### CLSB protocol and anesthetic technique

All CLSB procedures were conducted by the first author who was anesthesiologist with expertise in peripheral anesthesia. The patient was placed in the lateral decubitus position with the operated side uppermost and flexion of uninjured hip and knee, as much as possible. To provide patient comfort, a combination of midazolam (1-2.5 mg) and fentanyl (25-50 mcg) was given intravenously during positioning and needle penetration with oxygen supplement via nasal cannula flow 2-3 LPM. All peripheral nerve blockades were performed with nerve stimulator. Ultrasound guidance was used in the patients with a difficult anatomical landmark, such as obesity or spine deformity, or in those who had concomitant anticoagulant or antiplatelet medications to reduce risk of unnecessary iatrogenic trauma and bleeding complications. Lumbar plexus block (LPB) was achieved using the technique described by Capdevila^17^. In order to prevent the bleeding complication from anesthetic injection site, the patients with bleeding tendency, defined as having concomitant anticoagulant / antiplatelet medications or receiving thromboprophylaxis drugs, would receive only LPB with single shot technique using a small-sized (21-gauge) needle (Stimuplex® A Insulated needle 21G x 4’’, B Braun Melsungen AG, Germany). For those without bleeding tendency LPB with postoperative continuous infusion would be provided using a 18-guage insulated Tuohy needle (StimuLong Nanoline Tuohy tip 18 G x 50 mm. PAJUNK GmbH, Germany) to apply lumbar plexus catheter (StimuLong Sono-Tsui Set, PAJUNK GmbH, Germany). Initially, the needle was inserted and applied perpendicularly to the skin in all planes to contact the L4 transverse process of the operated side. The needle was then advanced 1-2 cm deeper to the transverse process level, either superiorly or inferiorly, by “walked off” technique. Generally, the total depth of needle penetration was usually about 5-8 cm from the skin. After that, an appropriate needle position was adjusted and confirmed, using nerve stimulator, as having a quadriceps contraction with a stimulating current of 0.4 mA. Next, a 20-ml mixture of 0.5% levobupivacaine and 2% lidocaine with epinephrine 1:200,000, in a ratio of 1:1 by volume, was injected slowly in aliquots. In the patients who had no risk of bleeding and planned for continuous LPB infusion, the catheter would be placed in a secure position away from operative side and then infused with 0.125% levobupivacaine, at a rate of 5 ml/hr., postoperatively. Then a sciatic nerve block was performed using the transgluteal^18^ or parasacral^19^ approach with the patient in the same position. The decision on techniques depended on patients’ injection site suitability. If the patients had abrasion or gluteal bed sore, the researcher would prefer to do parasacral approach. A 100-mm, 21 gauge insulated needle was inserted at a depth of approximately 4-6 cm. Foot plantar flexion or dorsiflexion was elicited with a stimulating current 0.4 mA. The same 20-ml local anaesthetic mixture was injected and the patient was then turned to the supine position. The onset time of the combined block was approximately 20-30 minutes. Therefore, a supplementary 10-ml local anaesthetics infiltration with 0.5% lidocaine with epinephrine 1:200,000 at the incision site was sometimes needed to allow some additional time for the block onset.

Postoperative pain control with oral medications (such as paracetamol or tramadol) and intravenous medications (such as fentanyl or morphine) were prescribed as *pro re nata* depending on the severity of postoperative pain. Postoperative mobilisation protocol was allowed for the patients to move the contralateral leg immediately after operation and to have sitting or upright position as tolerated without restriction. In the patients who received continuous LPB infusion, the catheters were removed 24 hours postoperatively in order to facilitate - early postoperative ambulation protocol.

### Data collection and presentation

Pre-operative patients’ demographic data, such as age, body weight, height, ASA status, presence of concomitant anticoagulant or antiplatelet medications, and underlying diseases, were recorded. Body mass index was further calculated. The patients’ cognitive function were assessed, preoperatively and postoperatively, using Thai Mental State Examination (TMSE) and diagnosed as cognition dysfunction or dementia if TMSE score ≤ 23^20^. Underlying diseases were categorized into hypertension, diabetes mellitus, anaemia, heart disease, respiratory disease, thyroid disease, renal disease, cerebrovascular disease, dementiaand autoimmune disease.

Perioperative data, as HF procedure, operation time, immediate postoperative complication related to anaesthesia, postoperative intensive care unit (ICU) requirement and duration of ICU stay, were collected. Immediate postoperative complication related to anaesthesia was defined as clinical presentations that may result from anaesthetic procedure or anesthetic drug used, as nerve injury, excessive bleeding at anaesthetic puncture site, hypotension following CLSB procedure, anaesthetic drug side effect and toxicity, incidence of GA conversion, and need for additional perioperative analgesia.

Postoperative data, such as analgesic medication used, duration of hospital stay, cognitive function status^20^, patients’ satisfaction and complications related to anaesthesia, mortality and morbidity related to HF, were recorded. Patient satisfaction was evaluated postoperatively before hospital discharge, using 10-point verbal analogue satisfaction rating scale (VASS, 0 = very dissatisfied with pain relief provided, 10 = very satisfied with pain relief provided)^21^, in all patients who did not have cognitive disorder.

## Results

A total of 70 high surgical risk geriatric patients were included into this study. Preoperative patients’ demographic data are presented in Table I. Fifty-three of them (76%) were female. The mean age was 78.3 ± 8.4 years (range 62-98 years). Thirty-three patients (54%) - had ASA IV status. There were 48 patients (69%) who had one or more anticoagulant medications. The most common co-morbid diseases were hypertension (40 patients, 57%), followed by heart disease (33 patients, 47%), diabetes mellitus and renal disorders (18 patients, 26%, both). There were 30 patients (43%) who received single shot LPB, while 40 patients (57%) received continuous infusion via lumbar plexus catheter.

**Table I:** Baseline Characteristics

Baseline Characteristics		n
Age (year)*		
	61-70	16 (23)
	71-80	25 (36)
	81-90	23 (33)
	91-100	6 (9)
Body weight (kg)◆		56 ± 11
Height (cm)◆		158 ± 8
Body mass index (kg/m2)◆		22.6 ± 3.9
ASA physical status*		
	III	32 (46)
	IV	38 (54)
Presence of anticoagulant therapy*		
	None	22 (31)
	aspirin	20 (29)
	aspirin and clopidogrel	9 (13)
	clopidogrel	9 (13)
	warfarin	4 (6)
	enoxaparin	6 (9)
Comorbid diseases*		
	Hypertension	40 (57)
	Diabetes mellitus	18 (26)
	Anaemia	16 (23)
	Heart disease	33 (47)
	Respiratory disease	8 (11)
	Thyroid disease	13 (19)
	Renal disease	18 (26)
	Cerebrovascular disease	12 (17)
	Dementia	11 (16)
	Autoimmune disease	3 (3)

* value presented as number of patients (proportion)

◆ value presented as mean ± standard deviation

ASA American Society of Anesthesiologists

Perioperative data and postoperative outcome are demonstrated in Table II. The most common HF operation was bipolar hemiarthroplasty (32 operations, 46%). The mean operative time was 3.2 hours (range 1.0–5.5 hours). None of patients required conversion to general anaesthesia. After the HF operations, all patients were awake with stable hemodynamic status, and able to move their contralateral leg. The mean arterial pressure was not significantly different during perioperative period (preoperative, intra-operative, and postoperative) (p >0.05, data not shown). All patients stayed in the post-anesthetic care unit only 30-60 minutes, and were transferred to ward without delay. There were nine patients (12%) who experienced discomfort as the immediate postoperative complication related to anesthesia, and the most common requirement was additional analgesics (5 patients, 7%), in the form of only small dosage of morphine (1-2 mg) or fentanyl (25 mcg). Three patients (4%), who had nausea and vomiting, were treated with one single dosage of intravenous ondansetron (4 mg). One patient who developed mild hypotension during the spinal block procedure was corrected with a single dose of intravenous epinephrine (5 mg) to restore his blood pressure. Thirty-six patients (51%) were admitted to ICU due to their medical conditions, and the majority of them (20 out of 36 patients) stayed in the ICU only one night. These statements do not correlate with Table II There were no cases of postoperative nerve injury, excessive bleeding or haematoma at puncture site, nor anesthetic drug toxicity.

**Table II:** Perioperative and Postoperative Outcome

**Perioperative data and postoperative results**		**n**
Operation*		
	Bipolar hemiarthroplasty	32 (46)
	Dynamic hip screw	12 (17)
	Proximal femoral nail	26 (37)
Total operative time (hour)◆		3.2 ± 1.0
Immediate postoperative complication*		
	Nausea and vomiting	3 (4)
	Mild hypotension	1 (1)
	Requirement of additional analgesics	5 (7)
Need of postoperative intensive care unit*		
	None	34 (49)
	1 day	20 (29)
	2 days	13 (19)
	3 days	1 (1)
	> 7 days	2 (3)
Postoperative analgesics requirements in the first postoperative 24 hours*		
	Morphine	34 (49)
	Tramadol	16 (23)
	Fentanyl	4 (6)
	Paracetamol	16 (23)
Postoperative hospital stay (day)◆		7.8 ± 6.9
Postoperative complications*		
	Death	1 (1)
	Delirium	1 (1)
	Sepsis with urinary tract infection	1 (1)
	Sepsis with pneumonia	1 (1)

* Value presented as number of patients (proportion)

◆ Value presented as mean ± standard deviation

During the first postoperative 24 hours, thirty-two patients (46%) required only simple analgesics, as paracetamol or tramadol (Table II). The mean dosages for intravenous analgesic were 4.1±2.0 mg for morphine, 31.3±12.5 mcg for fentanyl, and 73±17 mg for tramadol. The mean perioperative blood loss and total packed red cell transfusion were 205 ml (range 20-900 ml) and 0.4 units (range 0-2 units). There was no significant difference in the blood loss or transfusion requirement between patients with or without anticoagulant drugs (*p* > 0.05 both). The mean hospital stay was 7.8±6.9 days (range 4-60 days). During the hospital admission, one patient died at two months after operation due to - pneumonia and uncontrolled atrial fibrillation (inhospital mortality = 1.4%). There was one patient with urinary tract infection but recovered with antibiotics - and was - discharged 22 days after admission. One patient with pre-existing dementia developed postoperative delirium that was treated with medication by the psychiatrist. - (Table II). There was no symptomatic venous thromboembolic complication found in our study. All patients except those who had underlying dementia rated the VASS as 8 or more.

## Discussion

High surgical risk elderly patients undergoing hip fracture surgery require more specific attention due to their fragility. General anaesthetic options as general anaesthesia and neuraxial anaesthesia, that would result in higher complications due to - significant physiologic changes following those techniques with delay in post-operative recovery, might not be appropriate in this particular group of patients^14-16^. Therefore, peripheral nerve blockade with CLSB, which has a specific-site effect on only one leg and causes a lesser physiologic disturbance with a comparable anaesthetic effect, is possibly a more suitable alternative. However, through our knowledge, there were only a few case reports - that had provided the clinical outcome after using CLSB in high-risk geriatric patients undergoing HF surgery 15,16. Our retrospective study included as many as 70 HF patients with ASA physical status 3-4 who underwent HF surgery with CLSB we are able to report the outcome and safety of technique if performed by an experienced anesthesiologist.

Application of CLSB in HF surgery has several advantages. Firstly, CLSB has been proven as a safe and effective procedure in perioperative pain control as standard anesthetic method^14-16,22^. This is because the effect of CLSB could cover all innervated area of the hip and its joint capsule - supplied by lumbar plexus and branches of sciatic nerve^23^. Secondly, compared with NA, CLSB provided better hemodynamic control and avoidance of potential serious complications such as spinal or epidural hematoma, severe and prolonged intraoperative hypotension, and side effect from high opioid dosage^13,14^. Lastly, highly specific nerve blockade with CLSB, either single shot technique or continuous infusion via lumbar catheter, offered an immediate postoperative ambulation with high patient satisfaction. This is because CLSB has a sparing effect on the contralateral leg without the need for bed rest – protocol, resulting in the improvement of postoperative ambulationand the prevention of a number of serious complications, such as thromboembolism, myocardial infarction, and pneumonia^22, 24^.

In this study, the authors designed the use of a 20-ml mixture of rapid-acting agent with short duration of effect (10-ml of 2% lidocaine with epinephrine 1:200,000), and long-acting agent with long duration of effect (10-ml of 0.5% levobupivacaine) - which was different from the previous studies^14-16^. This - combined local anaesthetics (LA) solution would result in an early aneesthetic effect (approximately 20 minutes) which allow the anaesthesiologists to perform two blocks without delay without the need to use a high dose of levobupivacaine^25^, and also have a similar duration of analgesic benefit extended beyond six hours - comparable to the other studies^14-16^. Moreover, we also found that 20 mL of LA is sufficient for effective lumbar plexus block without serious complications like bilateral anesthesia (from unintentionally bilateral intrathecal/epidural drug spreading) or vascular administration of LA that had seen in traditional lumbar plexus block with large LA volumes (30-40 mL)^26^. Therefore, this proved that CLSB was a feasible option as a sole anesthetic technique for HF surgery in high-risk geriatric patients. However, until now, there is still inconclusive published data describing optimal dosage for LPB or sciatic block.

Based on the results of this study, CLSB demonstrated an excellent efficacy for perioperative and postoperative pain control without major anaesthetic-related complications, such as iatrogenic nerve injury, severe and prolonged hypotension, or contralateral leg blockade. These findings were comparable to the results of previous studies^14-16^. This present study also showed a comparable outcome on blood loss and blood transfusion requirement as seen in the previous studies^27,^ 28. However, due to the many possible factors that might affect the perioperative bleeding such as type of operations and the operative time, the authors could not conclude the exact relationship between early HF surgery in patients with or without anticoagulant therapy in this study. Additionally, we found that there was no puncture-site bleeding complication even in the patients who had bleeding tendency. This could be explained by our strict protocol that used ultrasound guidance during the needle application and use of a local anaesthetic mixture containing epinephrine. The overall minor anaesthetic-related complications were only 12% (mild hypotension 1%, nausea and vomiting 4%, need for additional analgesics 7%), which was reversible and curable with simple treatment. The in-hospital mortality rate in our study was very low (1.4%) compared to the previous study (30-day mortality rate = 17.4%)^29^ which may be explained by the effect of early surgical invention. Moreover, our study also demonstrated favorable clinical outcome as shown in a low need of ICU requirement (77% of patients did not require postoperative ICU or but only over-night ICU stay), and high satisfaction score. This supported the benefits of using CLSB with better hemodynamic control and postoperative ambulation.

Although this study showed a good outcome, we do not recommended it for routine CLSB use in HF surgery while NA with careful dose titration and closed monitoring is possible. This is because CLSB required two blockade procedures with large volume of local anesthetics which was technically more demanding, time-consuming, and the potential for toxicity. Therefore, CLSB should be considered whenever RA is strongly preferred but NA is contraindicated, such as for elderly patients with poor general health status and having bleeding tendency. Moreover, we also recommended using imaging guidance during needle application process in the patients with difficult anatomy or those having concomitant anticoagulant or antiplatelet therapy to improve accuracy and prevent the risk of potential complications such as bleeding at puncture site or and iatrogenic nerve injury^30^.

Our study has some limitations. First, this was a retrospective case series study, and further research is required to demonstrate the exact efficacy CLSB in HF surgery in general population. Secondly, although there was an excellent perioperative pain control, we also found one patient who had mild hypotension during the blockade procedure and this might relate to the local anesthetic dosage. Therefore, more studies may be required to find an optimal dosage for HF surgery to prevent this complication.

## Conclusion

The magnitude of the benefits of the CLSB is clinically important as a sole anesthetic for HF surgery, even in patients who have contraindications for NA or those with poor general health status. The greatest advantage of using CLSB as an alternative anaesthetic procedure in HF surgery is that it has very good efficiency and safety profile to operate on high-risk geriatric patients without any serious complications.
